# In Vitro Cell Death Mechanisms Induced by *Dicoma anomala* Root Extract in Combination with ZnPcS_4_ Mediated-Photodynamic Therapy in A549 Lung Cancer Cells

**DOI:** 10.3390/cells11203288

**Published:** 2022-10-19

**Authors:** Alexander Chota, Blassan P. George, Heidi Abrahamse

**Affiliations:** Laser Research Centre, Faculty of Health Sciences, University of Johannesburg, P.O. Box 1711, Doornfontein 2028, South Africa

**Keywords:** lung cancer, zinc phthalocyanine tetrasulfonate, photodynamic therapy, *Dicoma anomala*, apoptosis

## Abstract

Globally, lung cancer has remained the leading cause of morbidity and mortality in men and women. To enhance photodynamic therapeutic effects in vitro, the present study was designed to reduce dose-dependence in photodynamic therapy (PDT) and evaluate the anticancer effects of *Dicoma anomala* (*D. anomala*) root extracts (i.e., chloroform (Chl), ethyl acetate (EtOAc), and methanol (MeOH)) on A549 lung cancer cells. The most active extract of *D. anomala* (*D.A*) was used to establish the 50% inhibitory concentration (IC_50_), which was further used to evaluate the anticancer efficacy of *D.A* in combination with ZnPcS_4_-mediated PDT IC_50_. The study further evaluated cell death mechanisms by cell viability/ cytotoxicity (LIVE/DEAD^TM^ assay), flow cytometry (Annexin V-fluorescein isothiocyanate (FITC)-propidium iodide (PI) staining), immunofluorescence (p38, p53, Bax, and caspase 3 expressions), and fluorometric multiplex assay (caspase 8 and 9) 24 h post-treatment with IC_50_ concentrations of ZnPcS_4_-mediated PDT and *D.A* MeOH root extract. Morphological changes were accompanied by a dose-dependent increase in cytotoxicity, decrease in viability, and proliferation in all experimental models. Apoptosis is the highly favored cell death mechanism observed in combination therapy groups. Apoptotic activities were supported by an increase in the number of dead cells in the LIVE/DEAD^TM^ assay, and the upregulation of p38, p53, Bax, caspase 3, 8, and 9 apoptotic proteins. In vitro experiments confirmed the cytotoxic and antiproliferative effects of *D.A* root extracts in monotherapy and in combination with ZnPcS_4_-mediated PDT. Taken together, our findings demonstrated that *D.A* could be a promising therapeutic candidate worth exploring in different types of cancer.

## 1. Introduction

Cancer is a life-threatening disease affecting both men and women. It refers to a group of disorders in which the cancerous cells begin to proliferate and multiply in an uncontrolled manner [1]. If not treated, cancer can lead to the development of secondary complications and leads to death. Lung cancer is a common cancer that originates from the lung tissue, common in both men and women [2]. There are two major types of lung cancer, small-cell lung cancers (SCLCs) and non-small cell lung cancers (NSCLCs) [3]. According to the Global Cancer Observatory (GCO) 2020 to 2040 estimates, the global incidence rate of cancer is projected to increase from 19.3 million to 28.9 million cases by 2040. The global incidence rate of lung cancer is projected to increase from 2.21 million, as reported in 2020, to 3.63 million cases by 2040. On the other hand, the mortality rate is projected to increase from 1.80 million to 3.01 million cases. In Africa, the lung cancer incidence rate is projected to rise from 46 thousand to 92.2 thousand by 2040, while the mortality rate is projected to increase from 41.2 thousand cases to 82.8 thousand by 2040 [4]. The most common risk factors contributing to the increase in incidence and mortality rates include high alcohol intake, tobacco smoking, second-hand smoke, infections such as Human Papilloma Virus (HPV), radon exposure, and inflammatory diseases [5]. The most common signs and symptoms of lung cancer include shortness of breath, loss of appetite, cough, and fatigue [6]. Further, early detection of the highlighted signs and symptoms is essential in cancer therapy and management. Lung cancer is often diagnosed through physical, laboratory (e.g., blood biochemistry, sputum cytology, and pleural biopsy) and radiological examinations (e.g., ultrasound, computed tomography (CT), and X-ray) [7].

Current and common treatment modalities for lung cancer can be divided into two major classes, (a) localized and (b) systemic therapy. Common examples of localized therapeutic modalities for lung cancer include radiation therapy and surgery. This therapeutic approach is specifically applied to affected body parts. On the other hand, examples of systemic therapy include chemotherapy, hormonal therapy, and immunotherapy [8]. These treatment modalities can target both localized and metastatic cancers. Further, depending on the cancer type and its stage of progression, these treatment modalities may be administered individually or/ in combination with other therapeutic modalities, e.g., the combination of chemotherapeutic drugs with surgery. Although these therapies can induce tumor cell death, these therapies have been reported to induce undesired adverse side effects. Therefore, exploring novel, cost-effective, and well-tolerated therapeutic modalities would be ideal for addressing the limitations associated with conventional therapies for lung and other types of cancers.

Photodynamic therapy (PDT) or/ phototherapy is a novel therapeutic option employed in treating different types of cancer and certain non-malignant disorders [9]. In contrast to conventional therapeutic modalities, the mode of action behind PDT is mainly based on interactions of an excited state of a photosensitizer (PS) and molecular oxygen (O_2_), thus leading to the generation of cytotoxic reactive oxygen species (ROS), e.g., superoxide radicals (O_2_^•−^), hydroxyl radicals (^•^OH), hydrogen peroxide (H_2_O_2_) produced via type I photochemical reactions, and singlet oxygen (^1^O_2_) produced from type II photochemical reactions [10]. Despite its great potential in cancer treatment, PDT, as with any other therapy, has its shortcomings, e.g., low light penetration in large tumor masses. This is because visible light can only penetrate tissues between 5–10 mm in length [11]. In addition, the photothermal effect of PDT can also induce inflammation on the site of irradiation which eventually leads to the activation and induction of immunological responses [12,13]. Herein, we used a second-generation phthalocyanine PS: zinc phthalocyanine tetrasulfonate (ZnPcS_4_). Likewise, individual and combined effects of ZnPcS_4_-mediated PDT with an array of chemotoxic drugs (e.g., doxorubicin) and different anticancer plant extracts have been reported to induce tumor cell death [14,15]. To enhance the therapeutic efficacy of PDT, we used an active extract from *Dicoma anomala* (*D. anomala*/*D.A*) and combined it with ZnPcS_4_-mediated PDT in vitro. This is one of the effective therapeutic approaches for enhanced therapeutic outcomes.

*D.A* is a prostrate, herbaceous plant that belongs to the phylum Magnoliophyta, order Asterale, family Asteraceae. The leaves and roots of *D.A* have been reported to possess ethnomedicinal benefits in various medical conditions [16]. In tropical Africa, *D.A* is used as a herbal remedy for the treatment and management of medical conditions such as abdominal pains, dysentery, diarrhea, colds, coughs, sore throats, and sexually transmitted infections (STIs) [16,17]. Further, the anticancer activities of the *D.A* root extract isolate dehydrobrachylaenolide on breast, colon, melanoma, leukemia, and NSCLC cell lines have been summarized and reported in a comprehensive review by Maroyi [17]. With support from the above literature, we explored the anticancer effect of *D.A* crude extract and ZnPcS_4_ in monotherapy and combination therapy on A549 lung cancer cells in vitro. We further demonstrate that the IC_50_ for *D.A* crude extract and that of ZnPcS_4_-mediated PDT can trigger the activation of tumor cell death via the induction of different signaling pathways alongside the expression of apoptotic proteins. Finally, herein, we discuss the future prospects of *D.A* concerning clinical studies.

## 2. Materials and Methods

### 2.1. Collection, Identification, and Extraction of D.A

*D.A* was collected from the mountains of the Eastern region (13.6445° S, 32.6447° E) of Zambia. The Zambia Agriculture Research Institute (ZARI) verified the authenticity (phytosanitary certificate SR No: 0006064). Using cold tap water, the root of *D.A* was carefully cleaned before shade drying. Using a blender, the dried root was ground before being utilized in the Soxhlet extraction. Briefly, about 10 g of root powder was extracted in 70% of chloroform (Chl), ethyl acetate (EtOAc), and methanol (MeOH) for 36 h at 50 °C. For further studies, the extracts were stored at rtp in the dark. The stock solution of the extracts for experimental studies was prepared by dissolving about 10 mg (plant extracts) in 10 mL of 1× phosphate-buffered saline (PBS), thus giving a total concentration of 1 mg/mL. The experimental concentrations of root extracts were prepared by using the formula C_1_V_1_ = C_2_V_2_.

### 2.2. Cell Culture

The human lung cancer cell line A549 (ATCC^®^ CCL-185^TM^) from American Type Culture Collection (ATCC) was used in this study. The Rosewell Park Memorial Institute 1640 medium (RPMI) with 1% antibiotics (amphotericin B and penicillin/streptomycin) and 10% fetal bovine serum (FBS) was used for cell culture. Cells were incubated at 37 °C in an 85% humified incubator with 5% carbon dioxide (CO_2_) to 90% confluence. Afterward, the used complete medium was discarded, followed by a thrice wash with Hank’s balanced salt solution (HBSS) (10 mL). Experimental cells were detached from the culture flasks with TrypLE (Invitrogen, 12605-028) (3 mL). After that, experimental cells were cultured in 3.5 cm diameter culture dishes containing 2 mL of the complete medium at a concentration of 5 × 10^5^ cells/mL.

### 2.3. Photosensitizer (ZnPcS_4_) Co-Localization

The reagents, chemicals, protocol, and preparation of the ZnPcS_4_ stock solution used in this study are highlighted in our previous study [18]. To demonstrate subcellular co-localization of ZnPcS_4_ in A549 lung cancer cells, 3 × 10^5^ cells/mL were seeded on 3.5 cm culture dishes containing sterile microscope slides with complete pre-warmed RPMI culture medium (2 mL) and incubated for at least 4 h to 90% confluence. After which, incubated cells were washed thrice using 1× PBS and resuspended with complete RPMI media (3 mL). Washed cells were then treated with ZnPcS_4_ at the lowest concertation (5 μM) and incubated at 37 °C in an 85% humified incubator with 5% CO_2_. A549 cells were then washed three times using 1× PBS and fixed with 4% paraformaldehyde (1 mL) at rtp for 15 min. likewise, A549 cells were washed thrice using 1× PBS and permeabilized with 0.5% Triton X-100 (1 mL) for 15 min at rtp. Cells were then washed thrice using 1× PBS and suspended and incubated at 4 °C for 30 min with pre-warmed probes (200 μL), i.e., lysosomal tracker (Lyso-Tracker) (65 nM), endoplasmic reticulum tracker (ER-Tracker) (65 nM), and mitochondrial tracker (Mito-Tracker) (100 nM), sequentially. Following incubation, cells were washed thrice using 1× PBS, counter-stained with 200 μL of 4′,6-diamidino-2-phenylindole (DAPI) for 5 min, and then thoroughly rinsed three times using 1× PBS. Both control and experimental cells were then removed from the culture dishes, reinverted, and mounted with an aqueous mounting solution onto sterile glass microscope slides. After the slides were sealed with nail polish, DAPI, FITC, and Alexa Fluor 594 filters were used to check for ZnPcS_4_ organelle co-localization. This was done using a live imaging microscope, Carl Zeiss Axio Z1. Images were analyzed by using ZEN 3.1 (ZEN pro) software (Carl Zeiss, Göttingen, Germany).

### 2.4. Laser Parameters

A 680 nm semiconductor laser diode with a power output of 193 mW was used in ZnPcS_4_-mediated PDT. Post 4 h of PS incubation, A549 cells were irradiated using the laser parameters shown in Table 1, which was followed by 24 h incubation in a 37 °C, and 85% humified incubator with 5% CO_2_. All light-sensitive experiments of this study were performed in the dark, thus preventing light interference with PDT for accurate biochemical investigations. Before irradiation, the laser was turned on for 15 min, thus ruling out the possibility of laser instability. 

### 2.5. Dose-Response Studies

Dose responses were conducted on A549 cells to determine the optimal concentration from a more potent root crude extract of *D.A* and that of ZnPcS_4_-mediated PDT on A549 cells. The 50% inhibitory concentration (IC_50_) for both experimental models was determined by using trypan blue dye exclusion assay post 24 h treatment with varying concentrations of ZnPcS_4_ (5, 10, 20, 40, and 60 μM) and *D.A* root extracts (25, 50, and 100 μg/mL). Further, the active and more potent *D.A* root extract was used to determine possible phytocompounds.

#### 2.5.1. Morphological Assessment

Alterations in cellular morphology were analyzed using an inverted light microscope (Wirsan Olympus, CKX 41, Johannesburg, South Africa) with an attached digital camera (Olympus, C5060-ADUS, Johannesburg, South Africa) connected to CellSens imaging analysis software post-24 h treatment. Captured images of experimental models were then compared with control cells (untreated) for possible morphological changes.

#### 2.5.2. Trypan Blue Assay

The trypan blue dye (TPB) exclusion assay (Sigma-Aldrich T8154, Johannesburg, South Africa) was used to quantify the proportion of viable cells from non-viable cells in cell suspension from control and experimental cells. Briefly, about 10 μL of cell suspensions were mixed thoroughly to an equal volume of TPB (0.4%) (1:9). About 10 μL of the homogenous mixture was then pipetted on each window of sterile disposable countess^®^ slides. Cell viability was quantified by an automated Invitrogen counting II FL cell counter machine. The rationale behind the TPB exclusion assay is based on the ability of dead cells with cellular damaged membranes being unable to retain the TPB. Conversely, live cells with intact cellular membranes can retain TPB dye.

#### 2.5.3. Lactate Dehydrogenase Assay (Cytotoxicity)

The cell membrane integrity of A549 lung cancer cells was quantitatively analyzed by measuring the levels of cytosolic lactate dehydrogenase (LDH) released from A549 cells with distorted cell membranes. To assess LDH cytotoxicity, 50 μL of CytoTox 96^®^ Non-Radioactive Cytotoxicity assay (Promega, G179A) was added to an equal volume of cell culture medium, later incubated for 30 min at rtp in a 96 microwell plate and read using a Plate reader PerkinElmer, VICTOR Nivo^TM^ (Separation Scientific, Johannesburg, South Africa) at 490 nm.

#### 2.5.4. Adenosine Triphosphate Assay (Proliferation)

Adenosine triphosphate (ATP) proliferation assay was quantitatively assessed using CellTiter-Glo^®^ 3D luminescence (Promega, G968A, Anatech Analytical Technology, South Africa) kit on both control and treatment groups of A549 cells. This assay assesses the functionality of the mitochondria as well as the amount of ATP contained within the cells. Briefly, about 50 μL of CellTiter-Glo^®^ 3D luminescence was added to an equal volume of cell suspension in a 96 microwell plate. The mixture of ATP reagent and cell suspension was then mixed thoroughly and incubated at rtp in the dark for 10 min. Post-incubation, the resultant colorimetric mixture was analyzed for ATP luminescence using a plate reader PerkinElmer, VICTOR Nivo^TM^.

### 2.6. Combination Effects of D.A IC_50_ and ZnPcS_4_-Mediated PDT IC_50_

**Figure 1:**
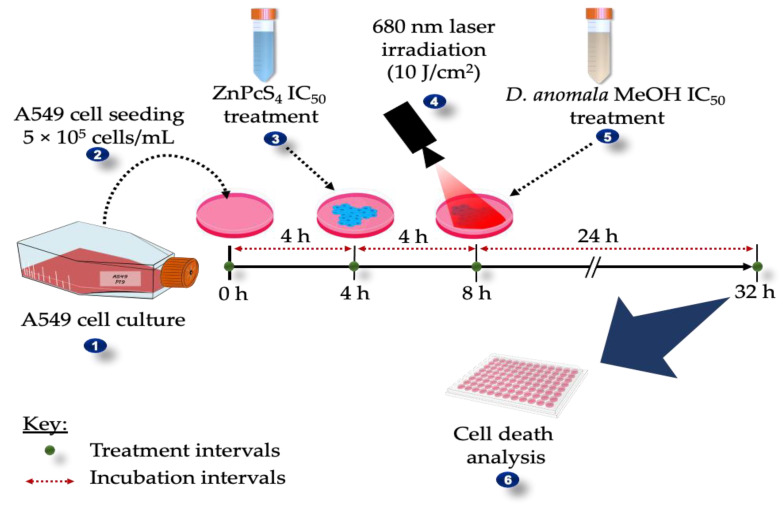
Schematic representation of treatment and incubation time. *D.A* MeOH IC_50_ was added to A549 cells post-treatment with ZnPcS_4_-mediated PDT and incubated for 24 h.

The combination effects were performed using the 50 % inhibitory concentrations (IC_50_) established from dose-response studies. In *D.A* treated A549 lung cancer cells, the IC_50_ was calculated from the more potent root extract. Since the study focused on the enhancement of ZnPcS_4-_mediated PDT, *D.A* IC_50_ was administered after treatment with ZnPcS_4-_mediated PDT. Post cell culture, the therapeutic schedules used to determine the outcomes from the combination of *D.A* IC_50_ and ZnPcS_4_-mediated PDT are depicted in Figure 1.

#### 2.6.1. Analysis of Cell Death

Likewise, morphological changes, cell viability, cytotoxicity, and proliferation assays were performed to evaluate the combined effects of the two IC_50′_s. In addition, to cell death analysis, cell viability/cytotoxicity (LIVE/DEAD assay), flow cytometry (Annexin V-FITC/PI), and immunofluorescence (p38, p53, Bax, and caspase 3 expressions) were performed post-24 h treatment with established IC_50′_s in monotherapy and in combination therapy.

##### Cell Viability/ Cytotoxicity (LIVE/DEAD^TM^ Assay)

The LIVE/DEAD^TM^ assay kit (Cat. No. L3224) (Molecular Probes) (Life Technologies Corporation, Johannesburg, South Africa) was used as per the manufacturer’s instruction to qualitatively visualize the distribution of viable and non-viable A549 lung cancer cells 24 post-treatment with ZnPcS_4_-mediated PDT IC_50_ and *D.A* MeOH root extract IC_50_ in both monotherapy and combination therapy.

Briefly, both untreated and treated cells were washed three times with ice-cold 1× PBS (1 mL), followed by resuspension of cells with 1 mL 1× PBS. Then, 1 µL of calcein, and 2 µL of ethidium homodimer-1 (EthD-1), were pipetted onto 3.5 cm diameter culture dishes containing A549 cells and later incubated for 30 min at rtp. Post-incubation, cells were washed thrice with 1 mL 1× PBS, resuspended in 1 mL 1× PBS, and visualized using Alexa Fluor 488 and EtBr filters under a Carl Zeiss Axio Z1 live imaging microscope.

##### Flow Cytometry (Annexin V-FITC/PI Assay)

Mechanisms of cell death (i.e., apoptosis and necrosis) were evaluated by flow cytometry using Annexin V-fluorescein isothiocyanate (FITC)-propidium iodide (PI) staining (BD Pharmingen™) as per manufacturer’s instructions. The rationale behind this assay is based on the interactions of annexin V-FITC/PI with the translocated phospholipid phosphatidylserine protein and nucleic acids, respectively. Post 24 h treatment, A549 cells were detached from culture plates by pipetting 300 μL of TrypLE™ onto the culture plates, which was followed by 5 min incubation at 37 °C. Once the cells were detached and suspended in a microtube, about 500 μL of ice-cold 1× PBS was pipetted into the microtube, and cells were later washed thrice by spinning at 2200 rpm for 4 min. The resulting pellet, after centrifugation, was resuspended in 1X binding buffer (500 μL). From the 500 μL cell suspension, 100 μL was pipetted into sterile flow cytometry tubes. To determine the cell death mechanisms, 5 μL of Annexin V and PI were pipetted into the sterile flow cytometry tubes containing 100 μL of cell suspension and incubated in the dark for 15 min. Post-incubation, 400 μL of ice-cold binding 1X buffer was pipetted into flow cytometry tubes containing 100 μL of cell suspension, 5 μL of Annexin V, 5 μL of PI, and incubated in the dark for 30 min. the evaluation of cell death mechanisms was then analyzed using Becton Dickinson (BD) Accuri^TM^ C6 flow cytometer. 

##### Immunofluorescence and Fluorometric Measurement of Caspase 8 and 9

To identify the possible cell death pathway that was activated, qualitative expression of cell death proteins was studied using immunofluorescence. In addition, the quantification of apoptotic proteins was determined by measuring the mean fluorescent intensity (MFI) of expressed proteins in the region of interest (ROI) and the MFI of the background. The final MFI of apoptotic proteins was determined using the formula: Final MFI = MFI of ROI—MFI of the background. Briefly, A549 cells were seeded onto 3.5 cm culture plates containing sterile coverslips. Following the treatment schedule illustrated in Figure 1, A549 lung cancer cells were treated with optimized concentrations, i.e., IC_50′_s of ZnPcS_4-_mediated PDT, and *D.A* MeOH root extract. Both untreated and treated cells were then incubated at 37 °C in an 85% humified incubator with 5% CO_2_ for 24 h post-incubation. The cells were washed thrice using 1× PBS and later fixed for 15 min at rtp by adding 4% paraformaldehyde (1 mL) into culture plates containing cells. Excess paraformaldehyde was washed out of the culture plates three times using an ice-cold wash buffer. Following a three-time wash, cells were then permeabilized for 15 min at rtp by adding 1 mL of 0.5% Triton X100 and washed thrice with 1× PBS. To avoid nonspecific binding of antibodies (Abs), about 1 mL of 1% Bovine Serum Albumin (BSA) was pipetted onto the culture dishes and incubated at rtp for 1 h. After incubation, cells were washed three times with 1× PBS.

Both primary and secondary Abs were prepared by following the manufacturer’s instructions. ThermoFisher Scientific (Johannesburg, South Africa) supplied the p38 MAPK (AHO1202), and Sigma-Aldrich (Johannesburg, South Africa) supplied the anti-p53 (SAB5700047), anti-Bax (WH0000581M1), and anti-caspase 3 (C8487) primary Abs were used in this study. About 200 μL of each primary Abs were added onto culture dishes and incubated in the dark at rtp for 2 h. After 2 h of incubation with primary Abs, cells were then washed three times with ice-cold 1× PBS to remove unbound Abs, treated by adding 200 μL of reconstituted fluorescent-labeled secondary Abs (i.e., rabbit anti-goat IgG-FITC (sc-2777), and goat anti-mouse IgG-FITC (sc-2010)) supplied by Santa Cruz^®^ Biotechnology. Cells were then washed three times with ice-cold 1× PBS post 1 h incubation, treated with 200 μL of DAPI for 5 min, and washed thrice with 1× PBS. After that, coverslips containing both untreated and treated cells were removed from the culture dishes and reinverted onto sterile microscopic glass slides with an aqueous mounting medium. After being sealed with nail polish, the slides were visualized using a Carl Zeiss Axio Z1 microscope. Using the filters DAPI, FITC, and Alexa Fluor 594, the slides were analyzed for the expression of apoptotic proteins using ZEN 3.1 (ZEN pro) software (Carl Zeiss, Germany). Further, captured images were quantified using ImageJ v1.53 software (National Institutes of Health, United States).

Additionally, the activities of caspase 8 and 9 were evaluated using a fluorometric multiplex activity assay kit (ab219915) (Abcam). The assay substrates were prepared by following the manufacturer’s instructions. 24 h post-treatment, A549 cell pellets were mixed with 100 μL of caspase 8 and 9 loading solution and incubated for 60 min at rtp. The fluorometric activities of caspases 8 and 9 were analyzed using a plate reader PerkinElmer, VICTOR Nivo^TM^. Caspase 8 was analyzed at wavelengths of 490 nm excitation/525 nm emission, while the excitation/emission for caspase 9 was set at 370 nm/450 nm. 

### 2.7. Statistical Analysis

Analyzed data from in vitro experiments were expressed as mean ± standard errors (SE). One-way analysis of variance (ANOVA) and the Dunnett test set at a confidence interval of 0.95 were used to determine statistical significance between treatment groups and control cells (untreated). Statistical significance between experimental groups is represented as *p* < 0.05 (*), *p* < 0.01 (**), and *p* < 0.001 (***). In vitro experiments were performed four times (*n* = 4), analyzed using IBM SPSS software v27, and graphs were plotted using OriginPro 2018 v9.5.1 software (OriginLab Corporation, Northampton, MA, USA).

## 3. Results

### 3.1. Photosensitizer (ZnPcS_4_) Co-Localization

**Figure 2:**
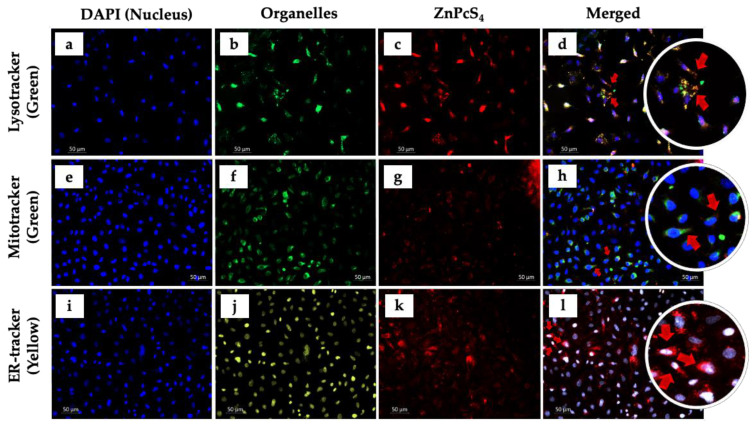
Intracellular co-localization of ZnPcS_4_ PS in A549 cells. Fluorescence micrographs of A549 cells showing blue fluorescence of DAPI (**a**,**e**,**i**), green fluorescence of Lysotracker (**b**) and Mitotracker (**f**), yellow fluorescence of ER tracker (**j**), red fluorescence of ZnPcS_4_ (**c**,**g**,**k**) and the merged image of ZnPcS_4_ and the organelle probes (**d**,**h**,**l**). (200× magnification and scale bar: 50 μm).

Intracellular co-localization of ZnPcS_4_ in A549 cells, stained with different organelle fluorescent tracker dyes and co-stained with DAPI, was visualized under a Carl Zeiss Axio Z1 microscope (Figure 2). As shown in Figure 2d,h, the red fluorescence (ZnPcS_4_) overlapped with green fluorescence (i.e., Lysotracker and Mitotracker probes). These results are indicative of intracellular co-localization of PS in the lysosomes as well as in the mitochondria. On the other hand, no organelle co-localization was observed in A549 ZnPcS_4_ treated cells, stained with ER-Tracker, and counter-stained with DAPI (Figure 2i–l). According to these results, ZnPcS_4_ does not co-localize within the ER of A549 cells.

### 3.2. Dose-Response Studies

#### 3.2.1. Morphological Assessment

**Figure 3:**
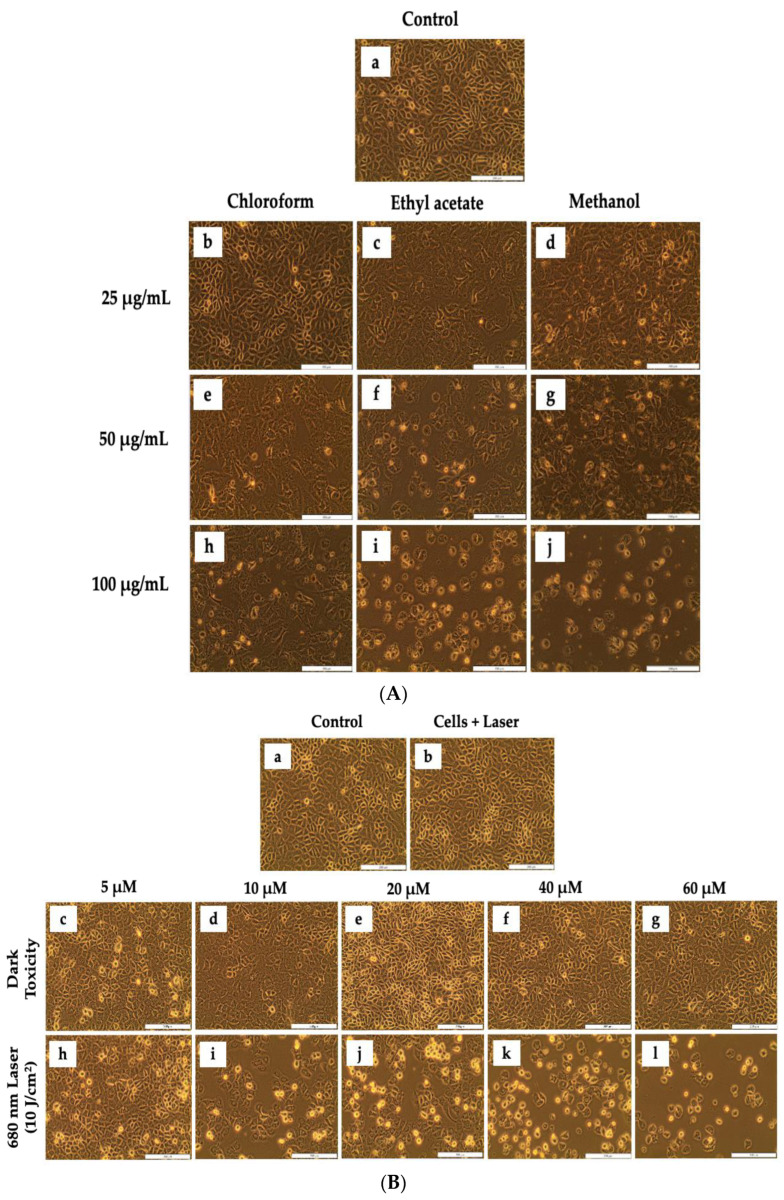
Morphological analysis of A549 cells post-treatment with *D.A* (Chl, EtOAc, and MeOH) root extracts (**A**) and ZnPcS_4_ dark toxicity and 680 nm laser irradiation (**B**). No morphological changes were displayed by A549 treated cells (**A**(**b**)) and (**B**(**b**–**g**)) when compared to the control cells (**A**,**B**(**a**)). Morphological changes were displayed by A549 cells (**A**(**c**–**j**)) and (**B**(**h**–**l**)). (200× magnification and scale bar: 200 μm).

Morphological analysis of untreated and experimental groups was conducted on A549 lung cancer cells treated with different concentrations of *D.A* root extracts (i.e., Chl, EtOAc, and MeOH extracts) and ZnPcS_4_ with and without laser irradiation (Figure 3). The untreated control cells and laser-irradiated cells showed a normal epithelial-like structure of A549 cells without any morphological alterations. Likewise, no morphological changes (i.e., rounding up of cells, vacuolization, detachment from culture dishes, and shrinkage) were observed in A549 cells treated with *D.A* Chl root extract (Figure 3A(b)), and ZnPcS_4_ without laser irradiation (dark toxicity) (Figure 3B(b–g)). However, morphological changes were observed in cells treated with different concentrations of *D.A* root extracts (Figure 3A(c–j)) and ZnPcS_4_ PDT at 10 J/cm^2^ (Figure 3B(h–l)). In addition, changes in morphology were observed in a dose-dependent manner. 

#### 3.2.2. Trypan Blue Assay

**Figure 4:**
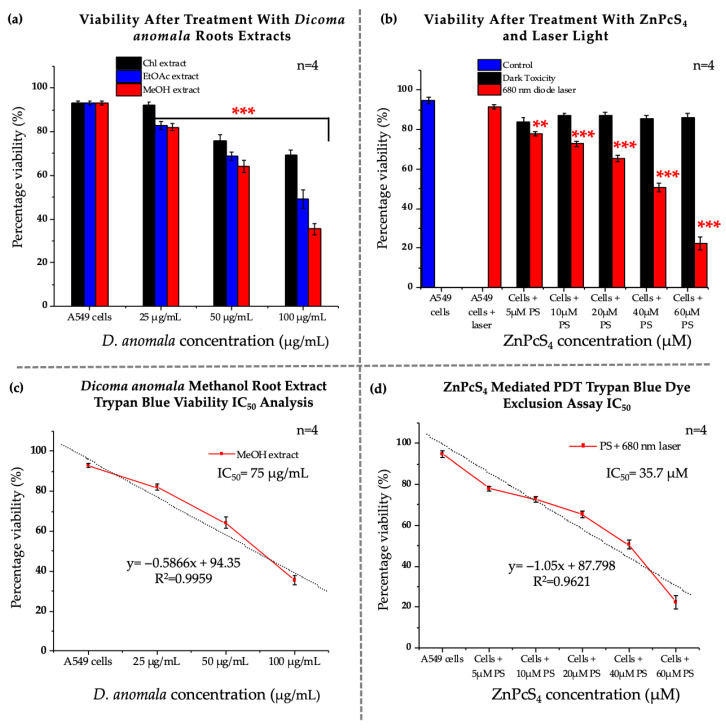
Trypan blue viability of A549 cells post-treatment with *D.A* (Chl, EtOAc, and MeOH) (**a**), and ZnPcS_4_ (**b**). *D.A* MeOH root extract IC_50_ (**c**), and ZnPcS_4-_mediated PDT IC_50_ (**d**). The values depicted in (**a**,**b**) are mean ± standard errors. Significant mean differences of independent treatments are represented as ** *p* < 0.01, and *** *p* < 0.001.

The percentage of metabolically viable cells from non-viable cells was determined by TPB cell viability assay post 24 h treatment with *D.A* root extracts and ZnPcS_4_. A549 cells treated with *D.A* Chl root extract (25 μg/mL) (Figure 4a) and ZnPcS_4_ (5, 10, 20, 40, and 60 μM) (Figure 4b) without laser irradiation showed no significant decrease in viability, whereas cells treated with the same concentration (25 μg/mL) of *D.A* root extracts (EtOAc and MeOH) showed a substantial decrease in cell viability when compared to the control cells. On the other hand, a significant decrease in viability was observed in cells treated with *D.A* in all A549 cells treated with *D.A* root extracts (i.e., Chl, EtOAc, and MeOH) at concentrations (50, and 100 μg/mL) (Figure 4a). Likewise, the same trends were observed in A549 cells treated with ZnPcS_4_ and later irradiated with laser light (Figure 4b). Based on viability results post-treatment with *D.A* root extracts, the MeOH root extract was more potent than the other extracts and was later used to determine the IC_50_ (75 μg/mL) (Figure 4c). Furthermore, the IC_50_ (35.7 μM) for ZnPcS_4_-mediated PDT was also determined by perfect linear fit (Figure 4d).

#### 3.2.3. Lactate Dehydrogenase Assay (Cytotoxicity)

**Figure 5:**
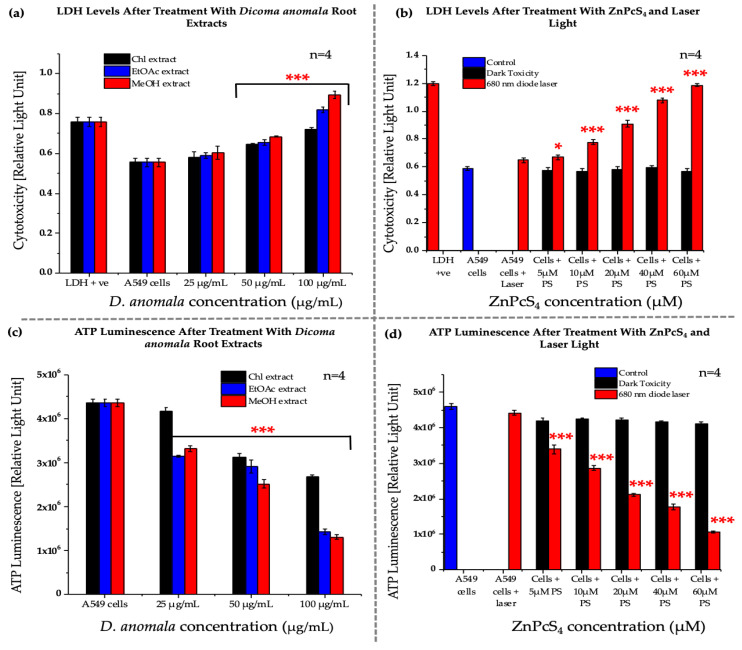
LDH cytotoxicity (**a**,**b**), and ATP proliferation (**c**,**d**) in A549 cells post-treatment with *D.A* (Chl, EtOAc, and MeOH) (**a**), and ZnPcS_4_. The values depicted in (**a**,**b**) are mean ± standard errors. Significant mean differences of independent treatments are represented as * *p* < 0.05, and *** *p* < 0.001.

LDH cytotoxicity was measured by measuring the amount of LDH released by A549 cancer cells post 24 h treatment with *D.A* root extracts and ZnPcS_4_. No significant increase in LDH levels was observed in 25 μg/mL *D.A* root extracts (Chl, EtOAc, and MeOH), ZnPcS_4_ (5, 10, 20, 40, and 60 μM)-treated cells without irradiation, and cells only treated with laser light when compared to the control cells. However, a significant increase in LDH release was observed in *D.A* root extracts (50 and 100 μg/mL) (Figure 5a) and ZnPcS_4_ (5, 10, 20, 40, and 60 μM) treated cells that received laser irradiation (Figure 5b).

#### 3.2.4. Adenosine Triphosphate Assay (Proliferation)

The energy levels of A549 cells were measured in both *D.A* (i.e., Chl, EtOAc, and MeOH root extracts) and ZnPcS_4-_treated cells post-24 h treatment. No significant decrease in ATP levels was observed in cells treated with laser, *D.A* (Chl extract) (25 μg/mL), and ZnPcS_4_-treated cells that did not receive laser irradiation (Figure 5a,b). However, a significant decrease was observed in *D.A* (Chl, EtOAc, and MeOH) root extracts (50 and 100 μg/mL) treated cells and ZnPcS_4_-mediated PDT when compared to control cells (Figure 5c,d).

### 3.3. Combination Effects of D.A IC_50_ and ZnPcS_4_ Mediated PDT IC_50_

#### 3.3.1. Morphological, Viability, Cytotoxicity, and Proliferation Analysis

**Figure 6:**
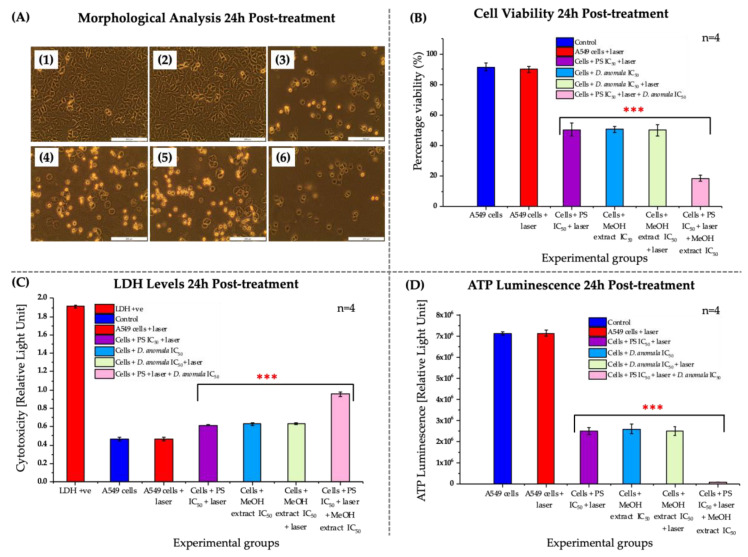
Morphological analysis of A549 cells post-treatment (**A**). Untreated cells (**A1**), 680 nm laser light treated cells (**A2**), ZnPcS_4_-mediated PDT IC_50_ treated cells (**A3**), *D.A* MeOH root extract IC_50_ treated cells (**A4**), *D.A* MeOH root extract IC_50_ + laser irradiation (**A5**), and *D.A* MeOH root extract IC_50_ + ZnPcS_4_-mediated PDT IC_50_ treated cells (**A6**). A549 cell viability (**B**), cytotoxicity (**C**), and proliferation (**D**). (200× magnification and scale bar: 200 μm). The values depicted in (**B**–**D**) are mean ± standard errors. Significant mean differences of independent treatments are represented as *** *p* < 0.001.

No significant morphological changes were observed in A549 cells treated with laser only when compared to control cells (Figure 6(A2)). However, significant morphological changes were observed in ZnPcS_4_ mediated PDT IC_50_, *D.A* IC_50_, *D.A* IC_50_ with irradiation, and the combination of the two IC_50′_s (i.e., ZnPcS_4_ and *D.A*) in A549 cells (Figure 6(A3–A6)). The TPB dye exclusion assay (viability) showed no significant decrease in cell viability in A549 cells that were treated with a 680 nm diode laser only, while a dose-dependent significant decrease in cell viability was observed in A549 cells treated with ZnPcS_4_-mediated PDT IC_50_, *D.A* IC_50_, *D.A* IC_50_ with irradiation, and the combination of the ZnPcS_4_-mediated PDT IC_50_ with *D.A* IC_50_ (Figure 6B). LDH release by dying cells into the culture medium was used to quantitatively evaluate the cell membrane integrity of A549 cells post-24 h treatment with established IC_50′_s. No significant increase in LDH release was observed in A549 cells treated with a 680 nm diode laser compared to the control cells. On the other hand, a significant increase in LDH release was observed in A549 cells treated with ZnPcS_4_-mediated PDT IC_50_, *D.A* IC_50_, *D.A* IC_50_ with irradiation, and the combination of the ZnPcS_4_-mediated PDT IC_50_ with *D.A* IC_50_ (Figure 6C). In addition, no significant decrease in ATP luminescence was observed in A549 cells that were treated with laser light only. However, a significant decrease in ATP levels was observed in cells that were treated with the two IC_50′_s in monotherapy as well as in combination therapy (Figure 6D).

#### 3.3.2. Analysis of Cell Death

##### Cell Viability/Cytotoxicity (LIVE/DEAD^TM^ Assay)

**Figure 7:**
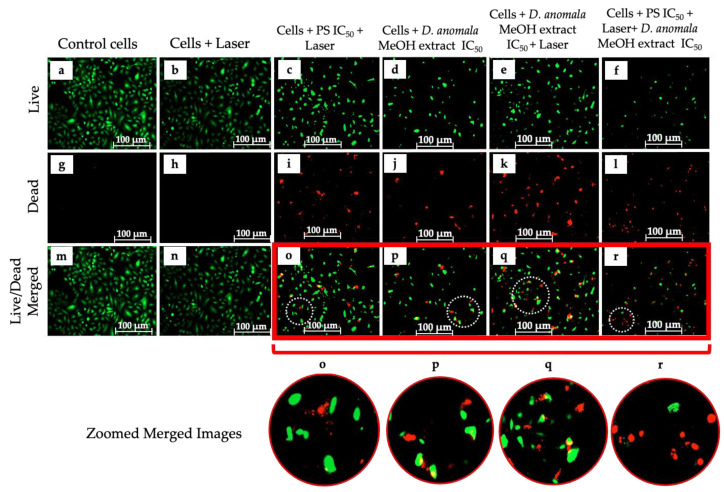
LIVE/DEAD^TM^ cell viability assay. Live cells stained green (**a**–**f**), dead cells (**g**–**l**), and the ratio of live to dead cells (**m**–**r**). (200× magnification and scale bar: 100 μm).

The LIVE/DEAD^TM^ assay was performed to qualitatively visualize the distribution of live and dead A549 cells post 24 h treatment with ZnPcS_4_-mediated PDT IC_50_, *D.A* IC_50_, and the combination of the two IC_50′_s (Figure 7). A549 cells stained with calcein (green fluorescence for live cells) and ethidium homodimer-1 (red fluorescence for dead cells). A549 live cells were stained green (Figure 7a–f), and dead cells stained red (Figure 7g–l). The number of live cells was higher in control cells and cells that received laser irradiation without the PS (Figure 7m–n), with more dead cells in IC_50_ treated cells (Figure 7o–r).

##### Cell Death Mechanism Analysis

**Figure 8:**
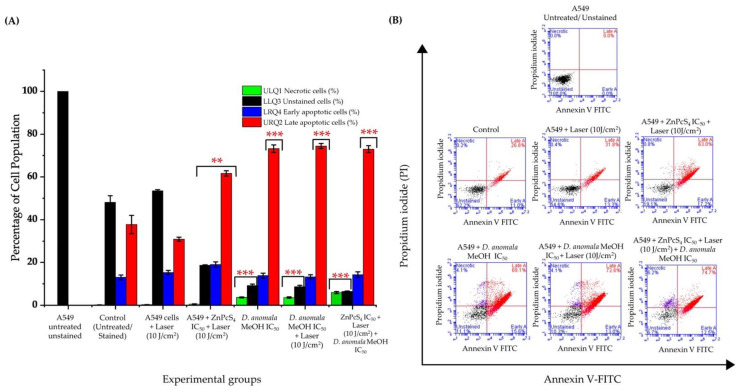
Flow cytometry results (Annexin V-FITC/PI) obtained in monotherapy and combination therapy (**A**) and scattergrams (**B**). The values depicted in (**A**) are mean ± standard errors. Significant mean differences of independent treatments are represented as ** *p* < 0.01, and *** *p* < 0.001.

Mechanisms of cell death were evaluated by Annexin V-FITC/PI staining in both individual treatment groups and combination therapy. Results from A549 unstained cells were used to gate the untreated and experimental cells. Laser-treated A549 cells at 10 J/cm^2^ showed no significant increase/or reduction in live/unstained and early apoptotic cells compared to control cells (Figure 8A). In monotherapy, live/unstained cells showed a significant decrease in the live cells population with a stronger significant decrease/or decrease in combination therapy. A Significant increase in late apoptosis was observed in both ZnPcS_4_-mediated PDT and *D.A* IC_50_ treated cells. It was also observed that early apoptosis was more favored than necrosis and late apoptosis in combination therapy. The schematic representation of one of the scattergrams obtained is shown in Figure 8B.

##### Immunofluorescence and Caspase 8 and 9 Analysis

**Figure 9:**
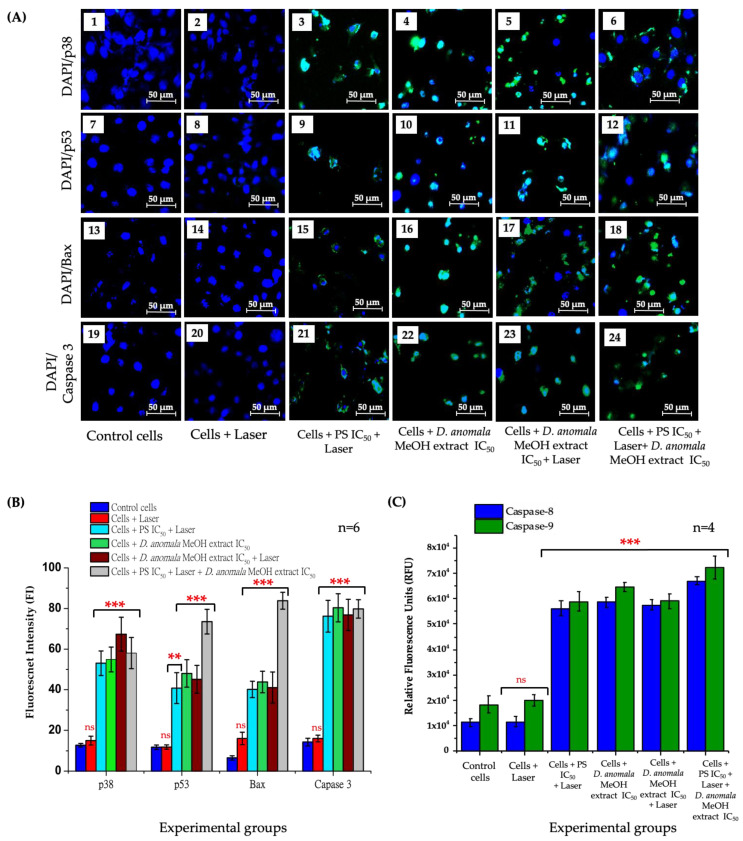
Apoptotic proteins (p38, p53, Bax, and caspase 3) visualization (**A**), mean fluorescent quantification (**B**) post-treatment with ZnPcS_4_-mediated PDT IC_50_ and *D.A* MeOH extract IC_50_, and the fluorometric multiplex activities of caspase 8 and 9 (**C**). Blue fluorescence (DAPI) stains the nucleus, and green fluorescence (FITC) represents the expression of apoptotic proteins. No expression of apoptotic proteins in A549 laser-treated cells (**2**,**8**,**14**,**20**). Increased expression of apoptotic proteins in A459 cells treated with ZnPcS_4-_mediated PDT (**3**,**9**,**15**,**21**), cells treated with *D.A* MeOH extract IC_50_ (**4**,**10**,**16**,**22**), A549 cells treated with *D.A* MeOH extract IC_50_ + laser irradiation (**5**,**11**,**17**,**23**), and cells treated with ZnPcS_4-_mediated PDT + *D.A* MeOH extract IC_50_ (**6**,**12**,**18**,**24**). (200× magnification and scale bar: 50 μm) (**A**). The values depicted in (**B**,**C**) are mean ± standard errors. Significant mean differences of independent treatments are represented as ** *p* < 0.01, and *** *p* < 0.001.

The activity of apoptotic proteins (i.e., p38, p53, Bax, and caspase 3) was qualitatively analyzed using Carl Zeiss Axio Z1 live imaging microscope and quantitatively analyzed using ImageJ analysis software post 24 h treatment with IC_50_ concentrations of ZnPcS_4_-mediated PDT and *D.A* MeOH root extract. DAPI and FITC channel filters were used to visualize the expression of the apoptotic proteins (Figure 9A), and mean mean fluorescent quantification (Figure 9B). No apoptotic protein activity was observed in cells treated with laser light only when compared to the control cells (Figure 9A(2,8,14,20)). However, the number of p38, p53, Bax, and caspase 3 positive cells was relatively high in cells treated with ZnPcS_4_-mediated PDT IC_50_ (Figure 9A(3,9,15,21)), *D.A* MeOH extract IC_50_ (Figure 9A(4,10,16,22)), *D.A* MeOH extract IC_50_ + laser (Figure 9A(5,11,17,23)), and in combination of the two IC_50′_S (ZnPcS_4_-mediated PDT IC_50_ + *D.A* MeOH extract IC_50_) (Figure 9A(6,12,18,24)). The quantification of the apoptotic proteins was significantly high in PS IC_50_ + laser, *D.A* MeOH extract IC_50_, *D.A* MeOH extract IC_50_ + laser, and the combination of the two IC_50′_s. However, no significant increase in apoptotic activities was observed in cells treated with laser light only for all experimental groups (Figure 9B).

Likewise, caspase 8 and 9 activities were evaluated by measuring the fluorescence of the control cells and the experimental groups (Figure 9C). No significant increase of both caspase 8 and 9 activities was observed in A549 laser-treated cells when compared to the control cells. However, a significant increase of caspase 8 and 9 was observed in ZnPcS_4_-mediated PDT IC_50_, *D.A* MeOH extract IC_50_, *D.A* MeOH extract IC_50_ + laser, and in ZnPcS_4_-mediated PDT IC_50_ + *D.A* MeOH extract IC_50_) treated cells when compared to the control cells.

## 4. Discussion

Despite major advances in current conventional treatment modalities, lung cancer has remained a major cause of cancer-related deaths among men and women [19]. This has resulted in a continual search for effective and alternative therapeutic strategies. Although PDT is considered a promising therapeutic option, a high dose is one of the major limitations associated with the treatment modality. However, to reduce dose dependence and completely eradicate cancer, in certain cases, cancer patients may require the administration of one or more therapies, e.g., PDT post-surgery [20]. Further, many plant-derived bioactive and secondary metabolites have been used to completely eradicate tumor cells in combination with PDT [21,22].

The visualization of intracellularly co-localized PS prior to PDT helps in identifying the specificity and location of the PS. It also helps in the determination and establishment of a comprehensive probable cell death pathway initiated post-PDT [23,24]. In addition to the aforementioned advantages of intracellular co-localization, the photophysical and photochemical properties (e.g., dimensional structures, partition coefficients, net charge, and lipophilicity) of PSs have been reported to influence the overall therapeutic efficacy of PDT [18,25]. It is also worth mentioning that the properties highlighted above play a crucial role in the transportation of drugs, e.g., a PS across the plasma membrane and eventually other organelles such as the lysosomes, mitochondria, and the ER [26,27,28]. These cellular organelles have distinctive physiological functions in tumor cell growth and destruction.

Lysosomes are subcellular membrane-bound organelles found in eukaryotic cells and contain hydrolytic enzymes that digest degraded cellular waste, thus promoting cell growth [29,30]. Interestingly, the enzymes contained within the lysosomes are synthesized in the ER (i.e., the rough ER) [31,32]. With regards to the mitochondria, it is a double membrane-bound organelle found in the cytoplasmic matrix of eukaryotic cells. The mitochondria is also regarded as the powerhouse of the cell because its function is to synthesize energy molecules in the form of ATP [33]. The mitochondria also plays a significant role in metabolic pathways such as beta-oxidation, pyruvate decarboxylation, ketogenesis, and apoptosis initiation accomplished via ROS generation and apoptotic proteins activation [34,35,36].

Unlike the ER, co-localization of the PS within the lysosomes and mitochondria is ideally essential in cancer PDT. This is because not only do these organelles perform their physiological functions, most of the intracellular ROS is generated within these organelles, thus making them a target for enhanced PDT [37]. In addition, increased ROS generation within the lysosomes may lead to lysosomal destruction, which eventually results in the release and translocation of hydrolytic enzymes (e.g., nucleases, glycosidases, sulfatases, proteases, phosphatases, and lipases). If released within the cytoplasmic matrix, hydrolytic enzymes may lead to the degradation of the major complex macromolecules (e.g., DNA, RNA, and lipids) of the cells [38,39]. With regards to the mitochondria, co-localization of PS within the organelle may lead to reduced production of ATP. Since ATP is needed for various cellular activities and processes to be initiated, depletion in ATP may result in the stoppage of vital cellular processes and eventually lead to cell death. It is also worth mentioning that cancer cells can generate about ~90% of endogenous ROS. One of the most common and vital cellular organelle involved in this process is the mitochondria. Additionally, imbalances and failure of cancer cells to eliminate the miochondiral generated ROS (mtROS) could lead to decreased antioxidative activities, thereby promoting oxidative stress on adjucent intracellular organelles [40,41].

In the present study, we evaluated the anticancer efficacy of the most potent root extract of *D.A* and used it to determine the IC_50,_ which was further used in combination with ZnPcS_4-_mediated PDT IC_50_. The findings of the present study demonstrated the therapeutic potency of *D.A* root extracts and ZnPcS_4_-mediated PDT on A549 lung cancer cells. It was also revealed that ZnPcS_4_ PS preferentially localizes in lysosomes, mitochondria, and the cytoplasm of A549 lung cells (Figure 2). The findings of the present study are similar to those of a previous study conducted by Manoto et al. [42], where it was revealed that sulfonated zinc phthalocyanine (ZnPcS_mix_), a mixture of sulfonated groups synthesized from hydroxylated ZnPcS’s, enhances the solubility of the PS, and preferentially localizes in the lysosomes and mitochondria of A549 lung cancer and human colon cancer (DLD-1) cells in vitro. Similarly, Tynga et al. [28] reported the intracellular co-localization of ZnPcS_mix_ in the lysosomes and mitochondria of MCF-7 breast cancer cells. Furthermore, intracellular co-localization of a PS within cellular organelles is reported to be crucial in the determination of possible cell death mechanisms [43]. 

During tumor cell death, tumor cells undergo a series of apoptotic morphological changes, including cell membrane blebbing, shrinkage, and rounding [44]. In the present study, these features were seen in cells treated with *D.A* root extracts (Chl, EtOAc, and MeOH) and ZnPcS_4_-mediated PDT, as well as in combination therapy (Figure 3 and Figure 6A). Although *D.A* is not intensively explored for anticancer effects, a study by Tripathy et al. [45] did demonstrate some of the features (e.g., tumor cell shrinkage) highlighted above on MCF-7 breast cancer cells post-treatment with silver nanoparticles synthesized from *D.A* Sond. root extract in vitro. Similarly, an in vitro study by Manoto et al. [46] reported dose-dependent morphological changes in A549 cells post-24 h treatment with ZnPcS_mix_-mediated PDT (680 nm) at a fluency of 5 J/cm^2^.

Recently, numerous studies have reported the cancer therapeutic benefits of herbal medicine [47,48,49,50]. In the present study, a dose-dependent decrease in cell viability, proliferation, and increase in cytotoxicity in *D.A* root extracts (Chl, EtOAc, and MeOH), and ZnPcS_4_-mediated PDT was observed in monotherapy (Figure 4 and Figure 5) as well as in combination therapy (*D.A* MeOH root extract IC_50_ and ZnPcS_4_-mediated PDT IC_50_) (Figure 6B–D) in vitro. These findings align with previous works from our lab [15,51], where different plant-derived bioactive compounds and plant crude extracts have been reported to decrease cell viability and proliferation. In addition, two possible active secondary metabolites (caffeoylquinic acid (CQA) and dicaffeoylquinic acid (di (CQA)) were identified by UHPLC-qTOF-MS^2^ in *D.A* MeOH root extract of our previous study [18]. In many studies, CQA and diCQA have been reported to possess anticancer, anti-inflammatory, and antibacterial properties [52,53,54]. A review by Abotaleb et al. [55] revealed that 1,3-diCQA could inhibit the proliferation of glioma cells and induce tumor cell death via ROS generation. With support from these studies, *D.A* MeOH root extract could be used to treat various cancers.

Additionally, apoptosis is a programmed cell death mechanism that plays a vital role in tumor cell death. This mechanism of cell death can be initiated by two pathways, the intrinsic or extrinsic signaling pathways [56,57]. Therefore, it is imperative to understand pathways induced by different therapeutic drugs employed in cancer therapy. Numerous factors have been found to play a crucial role in apoptosis induction, most of which are proteins in nature [58].

Subsequently, cell death analysis by Annexin V-FITC staining revealed two cell death mechanisms (necrosis and apoptosis) (Figure 8). Although two cell death mechanisms were detected in monotherapy and a combination of D.A MeOH root extract IC50 and ZnPcS4-mediated PDT-treated cells, apoptosis was a more predominant type of cell death seen in all treatment groups. A similar study by Aniogo et al. [59] reported apoptosis (early and late) being the most predominant cell death mechanism observed in MCF-7 breast cancer cells treated with the IC50 of doxorubicin (DOX) and ZnPcS4-mediated PDT. Similar observations were reported by Aydemir et al. [60], where hydroalcoholic extracts of *Ebenus boissieri* Barbey were used to treat A549 lung cancer cells in vitro. With support from the LIVE/DEAD assay (Figure 7), we strongly suggest that cells treated with IC_50_ concentrations of *D.A* MeOH root extract and ZnPcS_4_-mediated PDT showed early and late stages of apoptosis.

p38 mitogen-activated protein kinases are a group of proteins that belong to the class of mitogen-activated protein kinases (MAPKs). This protein plays a critical role in apoptosis and is stimulated by various factors (e.g., heat shock, UV irradiation, and proinflammatory cytokines such as IL-1 and TNF-α) [61]. Its function is to regulate multiple transcription factors. It has also been reported that the upregulation of p38 MAPK plays a significant role in the activation of mitochondrial caspase-mediated apoptotic cascade [62,63]. This initiation, in most cases, is initiated and promoted by the interactions of multidomain pro-apoptotic proteins (Bax and Bak) with the mitochondrial outer membrane, which eventually leads to mitochondrial outer membrane permeabilization (MOMP) and cytochrome c release in the cytoplasm [64,65]. Further, p53 is a tumor-suppressor gene that plays a critical role in DNA repair, regulation of the cell cycle, and apoptosis induction [66]. It has also been reported that p53 activities increase when a cell has damaged DNA and/or is under oxidative stress [67,68]. In response to DNA and oxidative stress, p53 accumulates into the cytoplasmic matrix, where it interacts with pro-apoptotic proteins, such as Bax and Bak, BH3-only pro-apoptotic protein initiators, and other members Bcl-2 family of proteins. These molecular interactions may directly or indirectly promote MOMP and tumor cell death by apoptosis [69,70].

**Figure 10:**
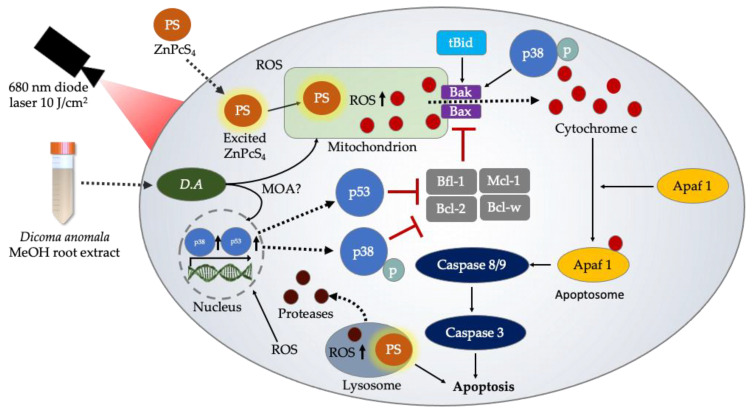
Schematic representation of the proposed intrinsic signaling pathways and cell death mechanism induced by ZnPcS_4_-mediated PDT and *D.A* MeOH root extract. ROS produced post-irradiation in the cytoplasmic matrix, mitochondria, and lysosomes together with *D.A* MeOH root extract chemotoxic effects lead to proteolytic and caspase-mediated cascade activation.

The present study presents evidence that the activity of apoptotic proteins (p38, p53, Bax, caspase 3, caspase 8, and caspase 9) in monotherapy and combination therapy promotes the induction of apoptosis. Furthermore, their interactions with intracellular organelles have been reported to promote the activation of apoptotic signaling pathways, e.g., the interactions of Bax with the mitochondria results in caspase cascade activation [64]. It was found that the activity of p38, p53, Bax, and caspase 3 was substantially increased in A549 cells treated with *D.A* MeOH root extract IC_50_ and ZnPcS_4-_mediated PDT IC_50_. Likewise, the activities of caspase 8 and 9 were significantly increased in *D.A* MeOH root extract IC_50_ and ZnPcS_4-_mediated PDT IC_50_ treated A549 cells. Additionally, it was observed that caspase 9 activities were slightly increased than that of caspase 8 in all experimental groups of the present study. This clearly suggests that the induction of apoptosis was intrinsically mediated. A similar study by Zhang et al. [71] reports the upregulation of p38 MAPK, p53, Bax, and clv-caspase-3,8,9 in A549 cells post-treatment with lobaplatin. Hence, our apoptotic protein expression results suggest the intrinsic apoptotic pathway is the key player in the induction of cell death in A549 lung cancer cells. Collectively, the results from our study pinpoint the proteolytic and caspase-mediated cascades induce apoptotic activities (Figure 10).

## 5. Conclusions

In conclusion, the anticancer potential of *D.A* root extracts against A549 lung cancer cells was evaluated in monotherapy and in combination with ZnPcS_4_-mediated PDT in vitro. *D.A* MeOH root extract induced cytotoxic effects that resulted in the upregulation of apoptotic proteins (p38, p53, Bax, caspase 3, caspase 8, and caspase 9) in vitro. Taken together, our findings revealed that *D.A* MeOH root extract is a promising candidate for the treatment of lung cancer and could be used in combination with novel therapeutic modalities such as PDT. In addition, this combination approach could possibly reduce the dose-dependence of various PSs, thus limiting the possibility of treatment resistance and cancer recurrence.

## Figures and Tables

**Table 1:** Laser parameters employed in ZnPcS_4_-mediated PDT.

Parameter Name	Description
Laser type	Semiconductor (Diode)
Wave emission	Continuous
Wavelength	680 nm
Spectrum	Visible light (Red)
Fluency	10 J/cm^2^
Output power	193 mW
Spot size	9.1 cm^2^
Irradiation time	8 min, 6 s

## Data Availability

Not applicable.
